# An integrated data pipeline for semantic data representation of the flame spray pyrolysis process

**DOI:** 10.12688/f1000research.161252.1

**Published:** 2025-02-07

**Authors:** Manuel Vollbrecht, Keno Krieger, Jannis Grundmann, Henk Birkholz, Norbert Riefler, Lutz Mädler

**Affiliations:** 1 Faculty of Production Engineering, University Bremen, Badgasteiner Str. 3, Bremen, 28359, Germany; 2 Leibniz-Institute for Materials Engineering, Badgasteiner Str. 3, Bremen, 28359, Germany

**Keywords:** Research Data Management, FAIR principles, application ontology, electronic lab notebooks, microservices, data acquisition pipeline

## Abstract

Ongoing digitalization and data-driven developments in materials science and engineering (MSE) emphasize the growing importance of reusing research data and enabling machine accessibility, which requires robust data management and consistent semantic data representation. Ontologies have emerged as powerful tools for establishing interoperable and reusable data structures from inconsistent data structures. Despite advancements in semantic data representation for specific applications, integrating application ontologies with primary data repositories, such as electronic lab notebooks (ELNs), to feed world data remains an open task. As a use case in the MSE domain, this work presents a system based on semantic technologies from the point of view of engineers, developed with the help of information scientists, and unraveled on a small scale. The development of an application ontology (AO) was elaborated for flame spray pyrolysis (FSP) processes with the implementation of a data pipeline. The proposed FSP application ontology emerges from experimental in-house best-practice procedures and is adapted to the mid-level Project Material Digital core ontology (PMDco) to allow interoperability within the MSE domain. The pipeline retrieves manually acquired experimental data from an ELN, translates it into a machine-actionable format, and converts it into a Resource Description Framework (RDF) format to support semantic interoperability. The latter was stored in a triple store with a SPARQL interface, enabling findable and accessible datasets that are searchable and traceable. By creating semantically linked data structures in line with FAIR principles, this approach allows traceable and findable experimental results between stakeholders through both human-readable and machine-actionable formats. Seamless integration of the modular microservices of the data pipeline within established lab practices minimizes disruption while maintaining the software framework. The present work demonstrates the practical implementation of a FAIR data pipeline within a laboratory setting, paving the way for future data-centric science.

**Abbreviations**
A-box
: Assertion box
AI: Artificial intelligence
AO: Application ontology
API: Application programming interface
ChEBi: Chemical Entities of Biological Interest
CSV: Character-separated values
dap: data acquisition pipeline
ELN: Electronic lab notebook
FAIR: Findable, Accessible, Interoperable, Reusable
FSP: Flame Spray Pyrolysis
HTML: Hypertext Markup Language
GUI: Graphical user interface
JSON: JavaScript Object Notation
LLM: Large Language Model
ML: Machine Learning
MSE: Materials science and engineering
NPO: NanoParticle Ontology
PMD: Platform Material Digital
PMDco: Platform Material Digital core ontology
PROV-O
: PROV ontology
T-box
: Terminology box
QUDT: Quantities, Units, Dimensions and Types
RDF: Resource Description Framework
SPARQL: SPARQL Protocol and RDF Query Language

## Introduction

Digital transformation in science and industry is driving the importance of data management and stewardship for data integration and reuse. ^1^ Research funders, publishers, and government agencies increasingly require robust data management plans for publicly funded projects [ 1, 2], emphasizing the need for findable, accessible, and reusable data. ^2^ In the course of Industry 4.0, companies thrive in the implementation of digital technologies such as artificial intelligence (AI) and machine learning (ML) to transform their production and supply chains into smart manufacturing systems. The ability to process first-hand data along diverse value chains is a critical prerequisite for data interoperability. ^3^ The FAIR principles (Findable, Accessible, Interoperable, Reusable) provide guidelines for achieving these objectives and supporting transparent research, reproducibility, and collaboration across diverse stakeholders within and beyond domains. ^2^ The effective utilization of knowledge and its representation within the Materials Science and Engineering (MSE) domain as well as industrial sectors is confronted with significant challenges owing to the inherent complexity, diversity, and interdisciplinary nature of the field. ^3^ Overlapping terminology and inconsistent data formats to the difficulty of aligning invariant and variant knowledge hinders seamless data-driven approaches. ^4^ These approaches are of great significance for the MSE domain, as they promise to accelerate material development with application-tailored properties ^5^ and pave the way for intelligent industrial production environments. ^6^

To overcome these challenges, the introduction of semantic web technologies has risen within the MSE domain, as they are capable of representing knowledge to be actionable and interpretable by machines while maintaining human readability. Specifically, ontologies are machine-readable representations of knowledge structured in Resource Description Framework (RDF) triples that have emerged as powerful tools in this context. ^7^ They enable seamless integration and flexible data retrieval, supported by query languages such as SPARQL. ^8^

Numerous national and international initiatives such as the National Research Data Infrastructure (NFDI) [ 3], National Institute of Standards and Technology (NIST) [ 4], National Institute for Materials Engineering (NIMS) [ 5], and European Materials Modelling Council (EMMC) [ 6] have evolved to push the development of ontologies by defining commonly shared data formats and providing open-access data repositories for enhanced collaborative ventures in MSE. The collaborative project Platform Material Digital (PMD) [ 7] aims to develop prototype infrastructures and tool solutions tailored for digital transformation in MSE. Central to these efforts is the concept of semantic interoperability, which ensures consistent data interpretation and exchange across platforms through the adoption of unified, scalable approaches, such as ontologies. To bridge the semantic gap between highly abstract top-level ontologies ^9, 10^ and specific application ontologies, the mid-level PMD core ontology (PMDco) provides abstract representations of central MSE concepts, thus facilitating the semantic description of MSE processes and material data and promoting interoperability and cross-domain collaboration efforts. ^4^ Building on the PMDco framework, MSE application ontologies have been developed and implemented, ranging from standardized tensile testing ^8^ and fatigue testing ^11^ to the development of functional inductors ^12^ and battery materials. ^13^

**Figure 1.**
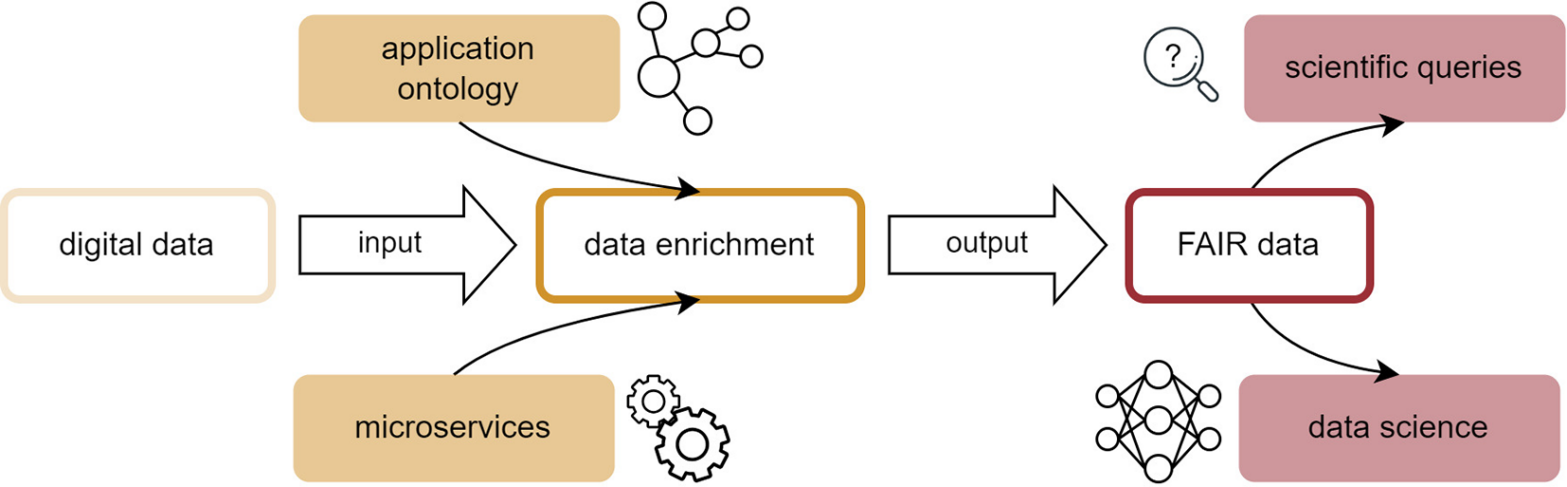
Data pipeline for the transformation of heterogeneous, non-uniform digital data originating from experimental processes into FAIR data structures.

The interconnection between an application ontology and an electronic lab notebook (ELN) is desirable for creating a seamless data pipeline from real-world experimental datasets to FAIR data structures. ^4, 8^ The present work represents a use case for this interconnection and introduces an application ontology for the flame spray pyrolysis (FSP) process as well as a data pipeline for associated acquired lab data initially aggregated in an electronic lab notebook, as shown in Figure 1.

## Methods

### Use case: Flame spray pyrolysis

Flame spray pyrolysis (FSP), equivalent to flame aerosol synthesis, ^14^ represents a versatile and scalable process for synthesizing metal oxide ^15^ and metal sulfide ^16^ nanoparticles for a broad range of applications, including gas sensing, ^17^ catalysis, ^18^ energy storage, ^19^ and medical applications. ^20^ The FSP process involves thermal decomposition of an atomized liquid precursor solution in the gas phase and the subsequent formation of solid particles through homogeneous nucleation and growth. Different process variants with methane- and hydrogen-fed flames, ^21^ single, ^22^ double flame set-ups, ^18^ open, ^22^ and closed reactors ^14^ have been reported for the synthesis of various tailored nanomaterials at laboratory and industrial scales. Lab-scale FSP reactors are characterized by individualized process layouts with varying degrees of automation and associated workflows, which pose challenges for consistent data management. The common experimental documentation of an FSP process comprises metadata, digital process (primary) data captured with measurement devices, and handwritten lab notes that essentially contribute to the reproducibility and traceability of reliable results. However, the lack of uniform experimental data inhibits efficient data integration and comprehensive reuse. In particular, highly individualized, non-standard experimental procedures that produce heterogeneous data formats require consistent data management to adhere to FAIR principles. In preparation for application ontology development, the in-house established FSP process and its associated workflow are introduced by means of an exemplary experiment in the following section. Furthermore, the role and use of the electronic lab notebook eLabFTW [ 8] for data documentation and the development of an MSE application ontology are presented.

### The process and experimental set-up

**Figure 2.**
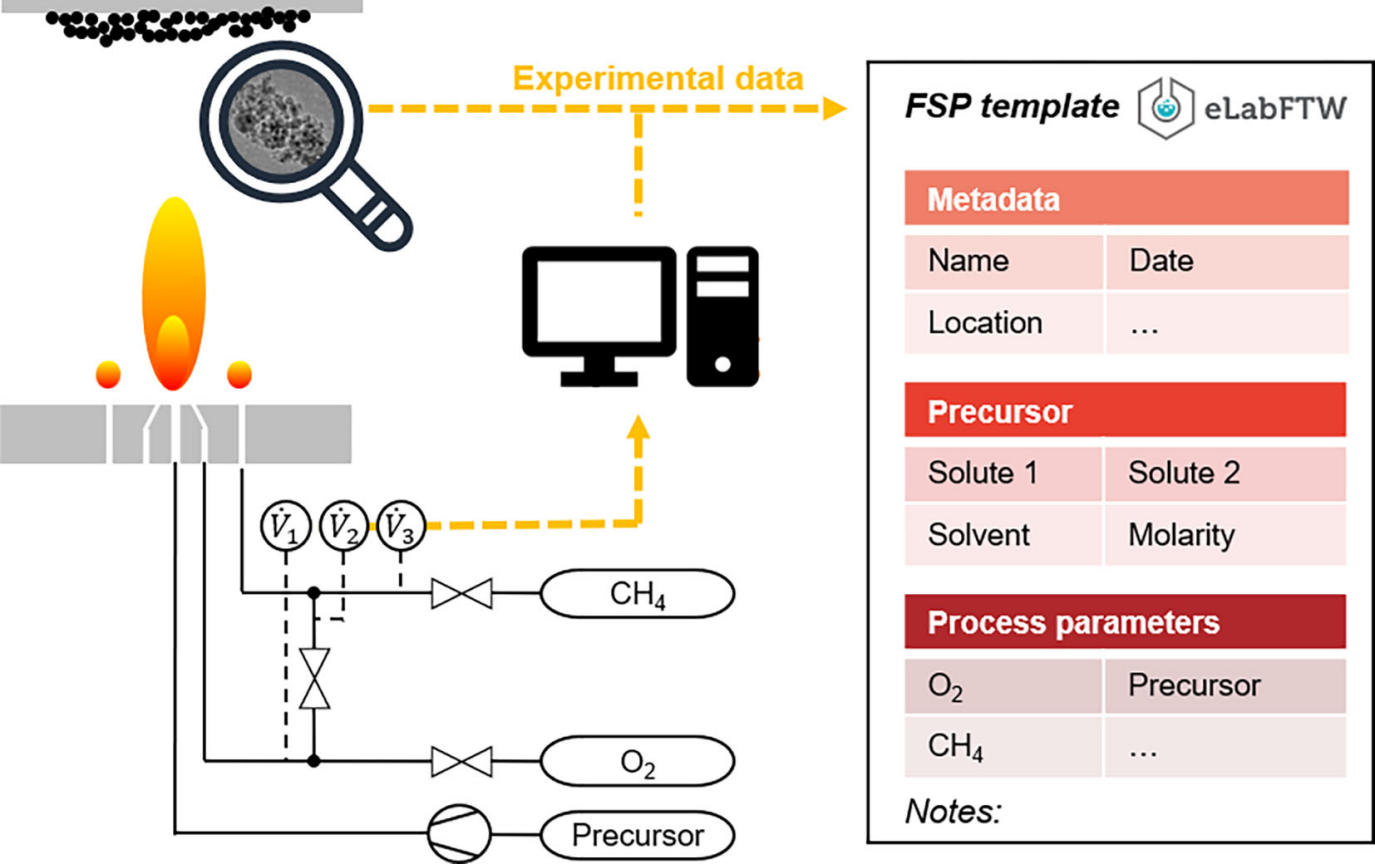
Schematic lab scale flame spray pyrolysis set-up including data acquisition using an electronic lab notebook (ELN) containing metadata, lab notes and in-process monitored data.

The present work refers to a specific single-flame FSP setup (cf. Figure 2), whereas the proposed framework is applicable to a broad range of FSP process configurations. The FSP experiment followed the general experimental sequence of (i) precursor selection and preparation, (ii) precursor atomization and spray combustion, and (iii) particle collection and subsequent characterization. Initially, an adequate combination of solvent and solute was selected for the preparation of the precursor solution. The solute (i.e., ferrocene) carried the desired chemical nanoparticle constituents (i.e., Fe ions), and the solvent (i.e., xylene) served as the dissolution medium for the solute, resulting in a liquid solution with the desired ion concentration. This precursor solution was fed through a capillary and underwent pressure-driven gas atomization, resulting in a fine spray. The atomized droplets are combusted in a high-temperature, self-sustaining flame supported by a surrounding pilot flame that is fed with fuel gas (e.g., methane) and oxidation gas (i.e., oxygen). As the spray passes through the flame, the droplets rapidly combust, leaving gaseous nanoparticle constituents and by-products of the combustion. The by-products form exhaust gases (CO _2_ and gaseous water), whereas the remaining free ions (Fe ions) react with the ambient oxygen-rich atmosphere and undergo simultaneous nanoparticle nucleation and growth. Nanoparticles (i.e., Fe oxide particles) are eventually separated from the gas stream and collected downstream using a suitable substrate (e.g., filter). All gas flows were controlled using gas flow controllers and a Labview ^®^ protocol with a graphical user interface (GUI) to specify the flow parameters.

## Implementation

### Acquisition of experimental data in an ELN

The size, composition, morphology, and material properties of the nanoparticles synthesized with FSP depend on principal process parameters such as precursor composition and concentration, as well as gas flow rates, as they influence the liquid-to-gas transfer of the precursor, ^23^ flame temperature, ^24^ and particle residence time in the flame. ^25^ Hence, a neat-less documentation of laboratory procedures comprising metadata and experimental specifications is essential to ensure the traceability and reproducibility of the results. The acquired experimental data were clustered manually and automatically into documented data. The latter includes the *online*-measured gas flow rates, which are stored in a *.csv* file as specified in the LabVIEW ^®^ routine. Data that require manual documentation comprise metadata and process settings, including precursor-related and process-related parameters, as well as specifications. In line with institutional data management [ 9], all experimental data were stored in the electronic lab notebook eLabFTW to gather different data formats in a single digital database entity to ensure consistent documentation. The use of an ELN facilitates efficient digital (meta) data integration and acquisition, along with enhanced data security and remote data access. ELNs also provide organizational benefits because experimental data, protocols, observations, and analyses can be structured and shared in a searchable format among teams, organizations, other affected groups, and nonacademic partners. An FSP-specific template was designed as a prerequisite for fully automated data retrieval ( Figure 2).

To ease digital data acquisition and transfer into the ELN, a simple Python flask webserver [ 10] with a RESTful [ 11] application programming interface (API) and graphical user interface (GUI) was implemented. This approach allows the experimenter to automatically create an ELN entry based on the experimental template, introduce metadata according to the DataCite [ 12] scheme through the GUI, and upload the acquired *.csv* file, which was the output of the experiment.

Webserver functionality further adds to process automation and facilitates communication with the ELN. After creating the ELN entry, the remaining primary data were manually added to the ELN entry. The uniform experimental documentation eventually enables fully automated data retrieval, connection to the application ontology, and thus the implementation of a fully integrated data pipeline. ^26^

### Development of the FSP application ontology

The development, implementation, and usage of ontologies in materials science applications provide exceptional benefits regarding data interoperability, structuring, and reuse based on a common shared domain understanding. By capturing fundamental concepts, associated entities, and the relations among them, application ontologies represent a semantic framework for modelling experimental workflows. Heterogeneous data can be transformed into consistent datasets, thus facilitating knowledge transfer and preservation across different domains. The development of an application ontology combines the efforts of both experimental and ontology-domain experts. Ontology development requires iterative steps: (i) process visualization, (ii) identifying principal process concepts and entities, (iii) their precise definition, including a taxonomy, (iv) a thesaurus, and (v) translating the process in the RDF-based web ontology language (OWL). Wherever possible, official norms and standards mark a reasonable starting point, as they already contain domain-agreed terms, including definitions. In the case of non-standardized experiments and workflows, such as the FSP process, the identification of principal concepts is based on a common, agreed-upon basis of domain experts. This early step in scoping and defining concepts and entities determines the detailed depth of the ontological model of the process. Concepts can be formulated from a bottom-up or top-down perspective. The first approach aims to capture the process from its fundamental entities onwards, which results in a highly detailed model. The high degree of detail poses challenges in aligning the ontology to related concepts of higher-ranked ontologies and, hence, leads to development efforts to account for inconsistencies. A top-down approach allows greater control of the detail depth; however, concepts may arise arbitrarily, resulting in extensive rework. A middle-out approach circumvents these disadvantages as the fundamental core concepts of a process are captured first, while the subsequent concretization of associated entities, that is, the detailed depth of the model, only evolves where necessary. ^27^ This step of ontology development can be efficiently aided by a process flowchart and mind mapping to cluster characteristic process entities and introduce an early taxonomic hierarchy. Subsequently, the semantic relations between concepts and entities are explicitly formalized in a thesaurus. The structured, well-defined vocabulary can ultimately be transferred to the ontology framework for machine-actionable semantic knowledge representation. Here, ontological concepts of existing (application) ontologies should be incorporated to allow data integration with domain knowledge. Semantic alignment with top-level and mid-level ontologies should be considered to facilitate data interoperability. The result of this transfer step was a terminology box (T-box). It can be understood as a structural model framework (i.e., classes) that describes the semantic link between actual processes and instances based on defined concepts and entities. Concrete data and knowledge derived from actual processes (i.e., an FSP experiment) are installed in an assertion box (A-box). The workflow of the FSP application ontology is outlined in the following sections.

### Capturing fundamental concepts

**Figure 3.**
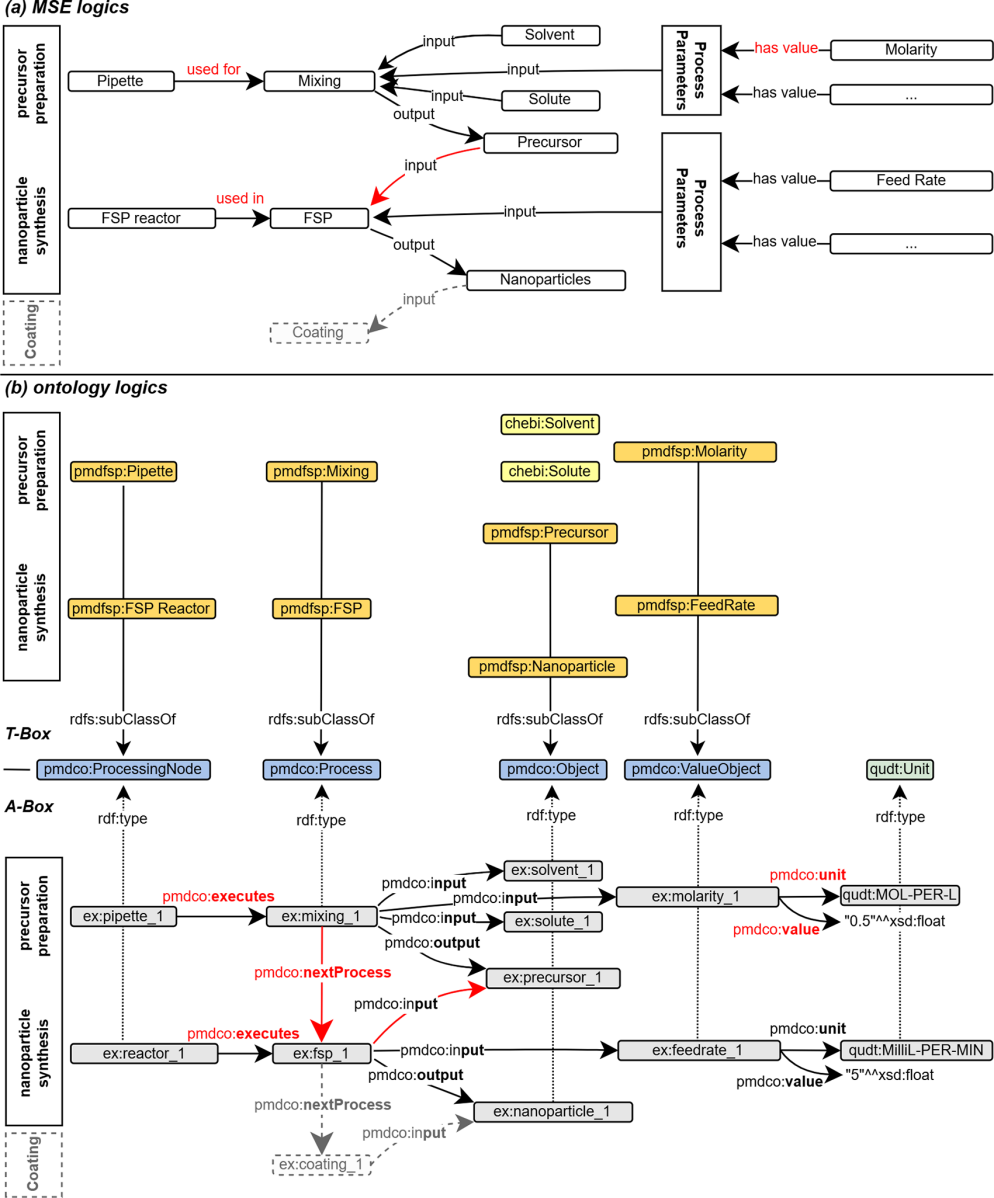
Comparison and alignment of the simplified FSP workflow with the mid-level PMD core ontology (namespace prefix *pmdco*): the MSE workflow (a) and associated classes (b) are aligned with the core classes of the mid-level PMDco highlighted in blue (T-box). The ChEBi (namespace prefix *chebi*) and QUDT (namespace prefix *qudt*) ontologies are incorporated in the FSP application ontology (namespace prefix *pmdfsp*) to use existing concepts of chemical entities and units, respectively. The A-box represents an exemplary FSP experiment (namespace prefix *ex*) with semantically linked instances of the respective ontology classes through corresponding *object properties.* In red are the different arrow directions in MSE logic compared to A-Box logic.

The first step involves abstraction of the introduced FSP process (cf. Figure 2) to formulate a conventional process flow chart, as shown in Figure 3a. The applied terminology is based on the in-house established FSP description as well as frequently used terminology in the corresponding literature. The experimental FSP workflow is broken down into a process chain of two subprocesses: (i) mixing of the precursor solution and (ii) the FSP process itself. Both processes require input such as physical items and (abstract) process parameters. In practice, precursor preparation is conducted with a pipette as the physical device to mix two chemical species (input) according to parametric specifications, such as molarity (input).

The sub-process outputs the precursor solution, which serves as the physical input for the FSP process, along with additional parameters. An FSP reactor is a physical device that conducts nanoparticle synthesis. The output of the FSP process is the nanoparticle product, which might serve as an input for subsequent processes (e.g., coating) and thus acts as a linking object to other processes. Naturally arising from MSE logic, the input arrows point towards the central process node, as it reflects the concrete working procedure. As anticipated, the process node points solely towards the output node. The abstraction of the FSP and construction of a workflow may be approached arbitrarily, resulting in different outcomes. Therefore, an early alignment with domain-specific ontologies and adaption of existing mid-level concepts is essential to ensure semantic interconnection, lower efforts in the ontology development process and its maintenance, and further promotion of consistency and standardization.

### Aligning with domain ontologies

The FSP application ontology (pmdfsp_ao) was designed to be fully adaptive with the PMD core ontology (PMDco, version 2.0.x) developed within the Platform MaterialDigital initiative as an extension of the PROV ontology (PROV-O) framework for semantic knowledge representation in the MSE domain. ^4^ The alignment of the core classes of the FSP ontology derived from the introduced experimental FSP workflow and underlying concepts with PMDco is shown in the form of a T-box in Figure 3b. The layout of the latter explicitly represents single processes or entire process chains. By inheriting the core PMDco [ 13] classes and their relationships (i.e., object properties), a framework for semantic representation of the experimental FSP process is readily available. Appropriate domain-specific ontologies are incorporated, where modelling requires abstract concepts beyond the workflow and process levels. The basic PMDco classes *ProcessingNode* and *Process* are used as parent classes to model the physical experimental assets (i.e., pipette and FSP reactor) used in the FSP process. Physical input and outputs (e.g., precursor solution, nanoparticles) as well as process parameters (e.g., molarity and feed rate) are defined in their respective *pmdfsp* classes as subclasses of the PMDco *Object* and *ValueObject* classes, respectively. For the formal semantic description of units and chemical entities of the precursor solution, the QUDT [ 14] and ChEBI [ 15] ontologies are inherited, respectively. Furthermore, the formal concept of nanoparticles is incorporated through NPO ontology [ 16]. The relationships, that is, object properties as established in the PMDco, between the entities of the proposed FSP ontology classes are shown in the A-box in Figure 3b. Two exemplary entities (namespace prefix *ex*) and the associated object property (denoted by the arrow) form a searchable data triple. This directed graph establishes a defined semantic representation of the two entities and their relationships, as highlighted.

### Serializing and validating the Ontology

Crafting an actual ontology requires the creation of a text file that contains the concepts of the ontology in a specific format based on the Resource Description Framework (RDF). RDF uses uniform resource identifiers (URI) to link and display the relationships between two virtual resources. This process is called serialization. For ontologies, different serialization formats exist, such as Turtle, JSON-LD, and RDF/XML, where data are stored in data triples. Inspired by natural language, these triples can represent a directed property graph and consist of a subject, predicate, and an object, with the predicate asserting a relationship between subject and object [ 17]. Essentially, the subject represents the entity being described, and the object contains the asserted subject property, which can be another URI-identifiable object or a literal value. An example of such a triple is the statement

The reactor|executes|the flame spray pyrolysis process.

or in the RDF syntax as derived from the A-box shown in Figure 3b:

ex:reactor_1 pmdco: **executes** ex:fsp_1. (1)

Here, the abbreviation before the colon denotes the namespace, that is, the ontological origin, of the subject, predicate, or object. Prefix ex is introduced to denote an example experiment that contains the respective ontological representation. The prefix pmdco refers to the PMD core ontology. ^4^ The PMDco defines general concepts for a semantic representation of processes in the MSE domain; for example, pmdco: **executes** from example (1). These concepts are represented by *classes* and their relations with each other, called *object properties.* The part of an ontology containing definitions for classes and their relations is often referred to as the terminology box (T-Box). This T-Box provides a collection of building blocks for modelling an experiment; for example, pmdco: Process, pmdco: Component, and pmdco: Sample. For a specific experimental setup such as the FSP, this collection needs to be expanded. This is achieved by defining new classes as *subclasses* of existing concepts using the rdfs:subclassOf object property, for example,

pmdco ManufactureProcess rdfs:subclassOf pmdco: Process

pmdfsp: NanoparticleSynthesis rdfs:subclassOf pmdco: ManufactureProcess

pmdfsp: FlamesprayPyrolyis rdfs:subclassOf pmdfsp: NanoparticleSynthesis

The fundamental advantage of defining new classes is *inheritance*: the new classes inherit all object properties associated with their ancestors (superclasses). The pmdco already contains a large set of object properties to model processes in material sciences and engineering, such as pmdco: **executes** from example (*) describing the relation between process nodes ( pmdco: ProcessNode) and processes ( pmdco: Process). Instead of defining these relations for each process from scratch, existing properties can be adopted by adding new subclasses to, in this case, Process and ProcessNode. Adding these new classes is the first step in assembling the application ontology (cf. Figure 3b T-Box).

A free and open-source tool for this task was the software *Protégé* [ 18] from Stanford University. ^28^ *Protégé* provides a graphical user interface for manipulating and saving ontologies in various formats. The triples contained in an ontology file are rendered in a manner that abstracts the complex underlying syntax and allows users to create new classes by right-clicking on existing classes. The hierarchies of the existing classes can be changed via drag-and-drop. Thus, *Protégé* lowers the entry barrier into ontology development and enables collaboration across scientific disciplines.

Modelling a real experiment requires assembling all building blocks from the terminology box (T-Box) into the assertion box (A-Box, cf. Figure 3b A-Box). The A-Box is populated with *individuals* (e.g., reactor_1 and fsp_1). These individuals are representations of, in this case, a real reactor and a real FSP process. Hence, the T-Box contains the theoretical framework for modelling an experiment, whereas the A-Box contains the actual experimental data. A- and T-Box are connected to the rdf:type object property. Every individual from an A-Box belongs to one or more classes from the T-Box (cf. Figure 3b).

A crucial step in ontology development is the re-assessment of the connections and relations drawn in the ontology. In the re-assessment, multiple aspects were considered:

(1) Is the ontology logic (formally) correct? Is everything well-defined?
(2) Does the ontology capture the fundamental features of the process? Does the ontology possess a suitable level of detail?
(3) Are the classes connected such that finding specific values or configurations is intuitive? Does the ontology design allow the capturing of process modifications?

Verifying the correctness of the formal ontology logic can be executed with a so-called *reasoner*, which is a program designed specifically for this purpose. Confirming that all definitions and relations are scientifically correct is a much more difficult task, as it requires the collaboration of multiple experts in the respective domain of MSE. This is especially difficult because relations between classes may be deliberately chosen differently as to how an expert would model the process for the usability purposes of the ontology. The applicability of the ontology for the desired use case should be the main focus of re-assessment. It is important that all the relevant process steps and parameters that can potentially impact the results of the experiment are included in the ontology. Here, one must strike a balance between keeping the ontology as simple as possible and including all important details of the experiment. Finally, the relationships between classes were examined. Conducting queries, that is, searching for experiments, can be performed based on an intuitive understanding of the process. Referring back to the examples above, a potential query can be:

What input did the flamespray pyrolysis have?

or, in the RDF query language (SPARQL)

SELECT ?input WHERE {

ex:fsp_1 pmdco:input ?input.

}

and should return ex:precursor_1. This aspect of ontology design is arguably the hardest, which will be addressed in the discussion of the pmdao_FSP layout below.

## Operation

### The pmdao_FSP ontology

The proposed application ontology (AO) aims to promote and establish FAIR principles for handling data retrieved during FSP experiments. The following section summarizes the key aspects of *pmdao_FSP* considered during ontology development.

### Key aspects of the pmdao_FSP

• **Wording according to commonly used terminology**

In the scope of FSP AO development, numerous concepts and classes required precise definitions that were predominantly dependent on in-house experience and wording. However, to allow for an intuitive understanding of class naming and its underlying definitions, the chosen terminology was aligned with domain-specific literature representing a common linguistic ground of potential users.

• **Applicable for various process configurations**

For in-house applications, both single-flame ^20^ and double-flame setups ^29^ were employed to synthesize tailored functional nanoparticles. These configurations utilize a wide range of liquid precursors, including diverse solvents, ^19^ metal organics, ^30^ and metal nitrates. ^31^ Moreover, various reactor configurations are used, including those based on fuel-fed ^17^ or hydrogen-fed flames. ^21^ Some processes incorporate different nozzle geometries, such as the Tethis nozzle ^22^ and SpraySyn nozzle, ^15^ whereas others utilize flame enclosures to tightly control the gas atmosphere during nanoparticle formation. ^16^ The proposed concepts and classes of the FSP application ontology (FSP AO) were designed to accommodate a wide variety of FSP process configurations. This flexibility enhances its applicability to third-party FSP users, facilitating the broader adoption of FAIR data practices in the field.

• **Addressing common process challenges**

The FSP ontology concepts and abstractions were developed in accordance with best practices to address prevalent challenges in the flame spray pyrolysis (FSP) process. A notable example concerns precursor chemistry, where ensuring the solubility of diverse metallic ion species necessitates careful selection of appropriate precursor-solvent combinations. The frequent lack of negative experimental results renders such data largely inaccessible, thereby impeding knowledge transfer and reuse by third parties. This underscores the necessity of implementing FAIR research data, which is facilitated in this ontology through the use of searchable data triples. The class definitions within the FSP ontology are specifically designed to inherit the object properties from the PMD core ontology (PMDco), thereby enabling seamless data interoperability. This structure also enhances the user-oriented searchability of information, particularly for recurring questions related to the experimental procedures. Through this approach, the ontology supports efficient access to critical data, ensuring a robust foundation for knowledge sharing and the promotion of FAIR principles in the FSP research domain.

• **Adaption to PMD core ontology (2.0) and popular domain ontologies**

**Figure 4.**
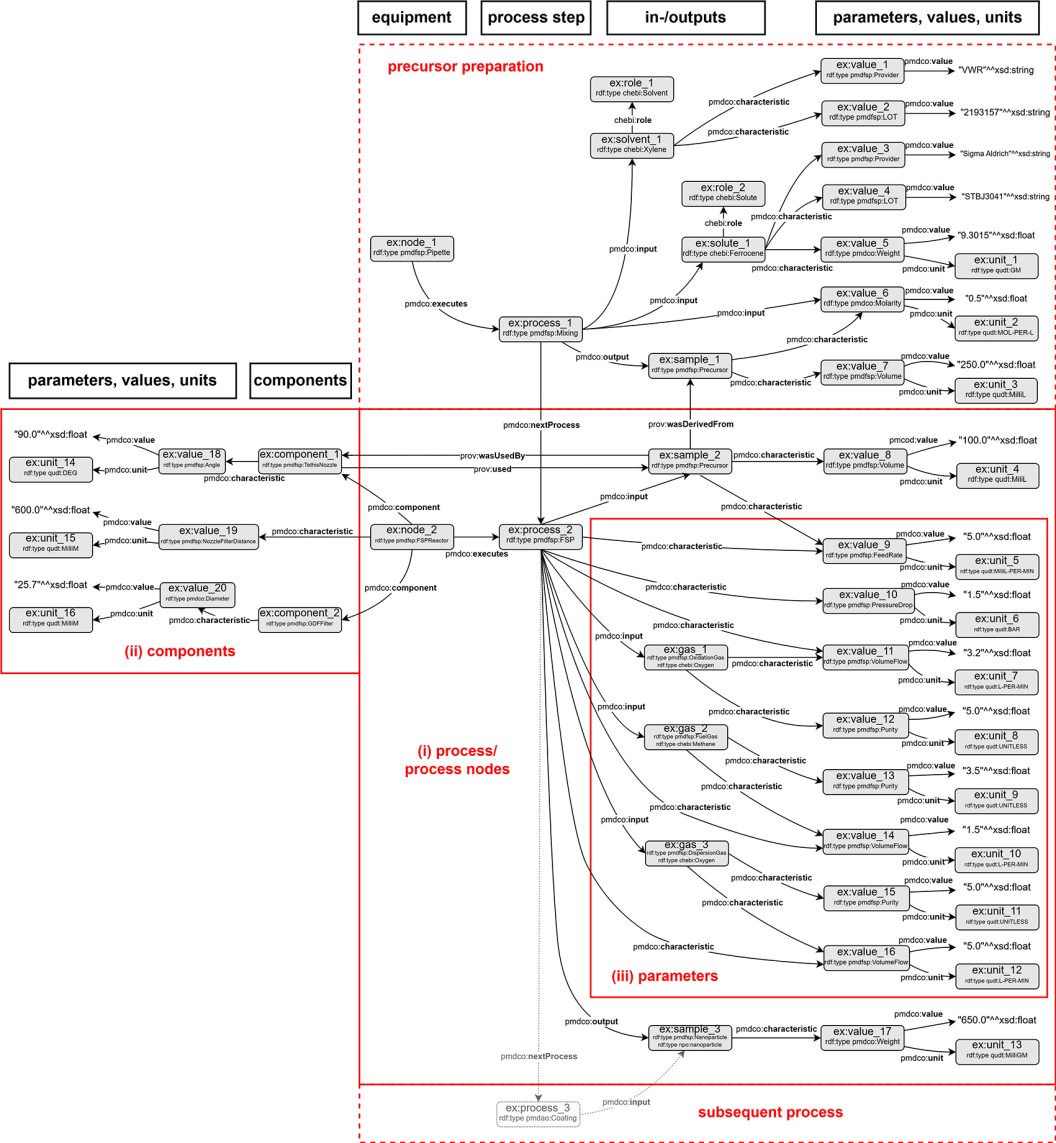
Assertion box of an FSP experiment displaying exemplary entities (grey) of the respective *pmdao_fsp* (namespace prefix *pmdfsp*) classes. Linked entities with assigned *object properties* form searchable data triples. The *object properties* are inherited from the PMDco (namespace prefix *pmdco*) mid-level ontology.

The application ontology is designed to be adapted to the PMD core ontology (PMDco), which links specific ontological applications in materials science and engineering (MSE) with overarching top-level ontological concepts. By integrating standardized mid-level ontological concepts, the ontology fosters data interoperability within the flame spray pyrolysis (FSP) domain and across related fields. As depicted in Figure 4, this adaptation enables the modelling and semantic integration of subsequent processes that leverage the outputs of the FSP process, specifically the synthesized nanoparticles. Such capabilities are essential to facilitate the interdisciplinary reuse of domain-specific data, thereby enhancing the potential for collaborative research and knowledge transfer.

• **Documented and openly accessible**

**Figure 5.**
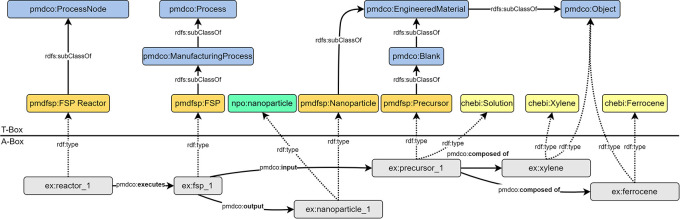
Principle entities displaying the workflow of the introduced FSP process and the inheritance of basic *pmdco* classes (cf. Figure 4 (i)). An FSP reactor ( *pmdco: ProcessNode*) executes an FSP process ( *pmdco: Process*) which receives an input entity (precursor, *pmdco: Object*) and provides nanoparticles ( *pmdco: Object*) as process output.

The AO is documented and openly accessible to Github [ 19]. The repository further lists the eLabFTW template for the documentation of individual FSP experiments and five exemplary experimental datasets, including their semantic representation as an A-box ( *.ttl* ). The latters are used to demonstrate the scientific querying and potential of targeted data retrieval from numerous datasets (cf. Section **From lab notes to structured FAIR data**). The open documentation of the FSP AO assists the community in engaging in its further development through encouraged collaboration and refinement of underlying concepts.

### Layout of the pmdao_FSP

The following sections highlight the ontological modelling of the core FSP AO concepts and the adaption of the hierarchical class structure of PMDco. These sections represent single excerpts from the entire exemplary A-box shown in Figure 4.

### Modelling of general FSP process

An FSP experiment was abstracted using the class *pmdfsp: FSP* as a subclass of *pmdco: ManufacturingProcess* and *pmdco: Process.* The FSP reactor ( *pmdfsp: FSP Reactor*), which is a subclass of the process node class ( *pmdco: ProcessNode*), employs the object property, *pmdco:executes* which semantically links a defined reactor layout (as described above) with a specific FSP experiment. The process itself semantically receives physical entities (e.g., precursors) as input and outputs nanoparticles through the object properties *pmdco:input* and *pmdco:output*, respectively. Physical entities are modelled as instances of the respective classes being a subclass of the *pmdco: Object* class. Thus, the composition of the precursor solution ( *chebi: Solution*) can be expressed as a combination of solvent and solute, which can be individually searched through the *pmdco:composed of* object properties.

### Modelling of reactor

**Figure 6.**
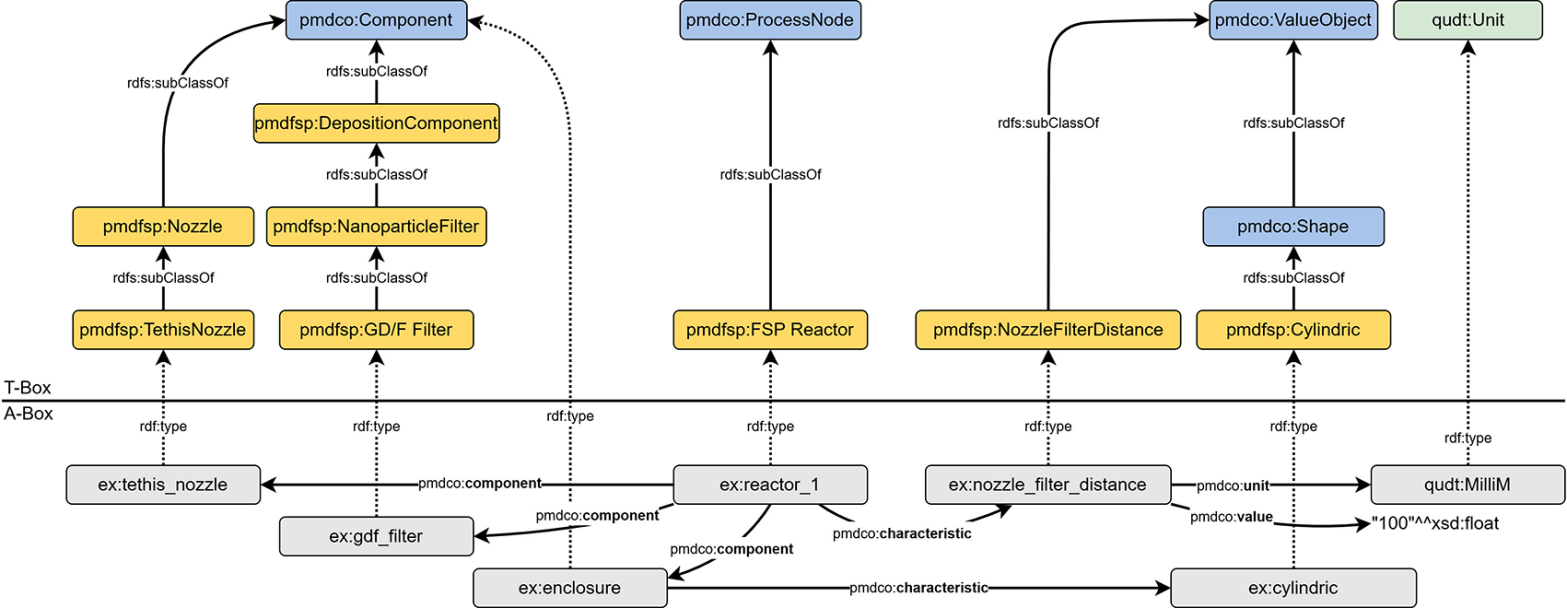
Semantic representation of an exemplary FSP reactor (cf. Figure 4 (ii)) consisting ( *pmdco:component*) of a tethis nozzle, a filter as deposition medium and an enclosure ( *pmdco: Component*). The distance between the filter and the nozzle is specified through the respective parameter ( *pmdco: ValueObject*) given conventionally in millimetre ( *qudt: Unit*).

The reactor configuration associated with an FSP experiment can be represented semantically using the data triples shown in Figure 6. The nozzle, filter, and optional enclosure are instances of subclasses of *pmdco: Component.* In this way, the process node (i.e., the FSP reactor) can be characterized through its abovementioned components and the *pmdco:component* object property. Specifications of both components and process nodes are incorporated as value objects linked with *pmdco:characteristic* properties of pmdco.

### Modelling of process parameters

**Figure 7.**
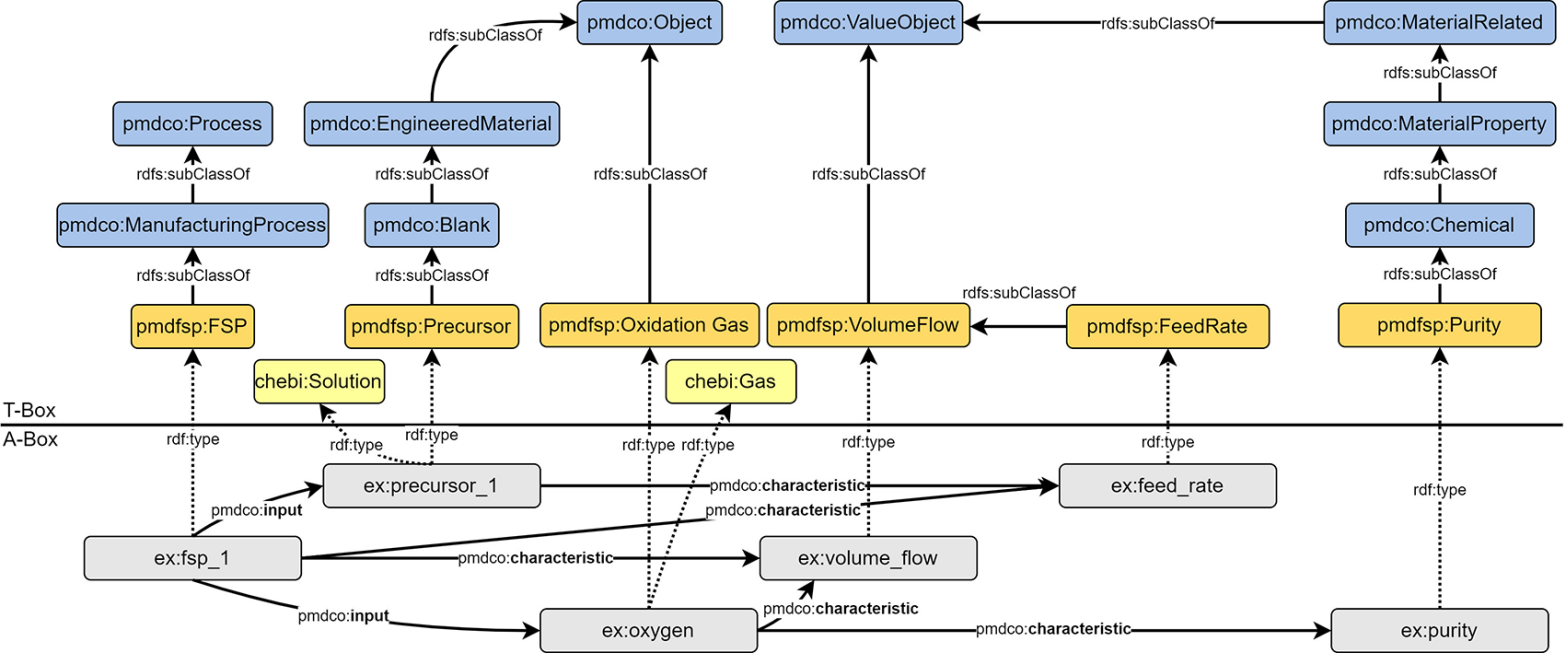
Semantic representation of precursor and oxygen gas as exemplary process inputs (cf. Figure 4 (iii)) including respective specifications ( *pmdco:characteristic*). Process parameters such as volume flows ( *pmdfsp: VolumeFlow*) and purity ( *pmdfsp: Purity*) are defined as subclasses of the generic *pmdco: ValueObject* class and directly related to the FSP process ( *pmdfsp: FSP*).

FSP parameters are generally subclasses of the *pmdco: ValueObject* class. The respective instanced entities are semantically linked to both the process entity ( *ex:fsp_*1) and their object entities. This simplifies the search for information regarding specific process characteristics. Figure 7 shows an example of how domain-specific terminology is incorporated into the design of the AO and the definition of new classes. From a physical point of view, all gases and the liquid precursor are fed into the process ( *pmdco:input*) and specified as the temporal volume flow. However, for the precursor, the term “feed rate” ( *pmdfsp: FeedRate*) is mostly used. Thus, inheriting domain-specific terms in the definition of new classes promotes an intuitive understanding of semantic FSP representations.

### Data ontologization using microservices

**The framework**

**Figure 8.**
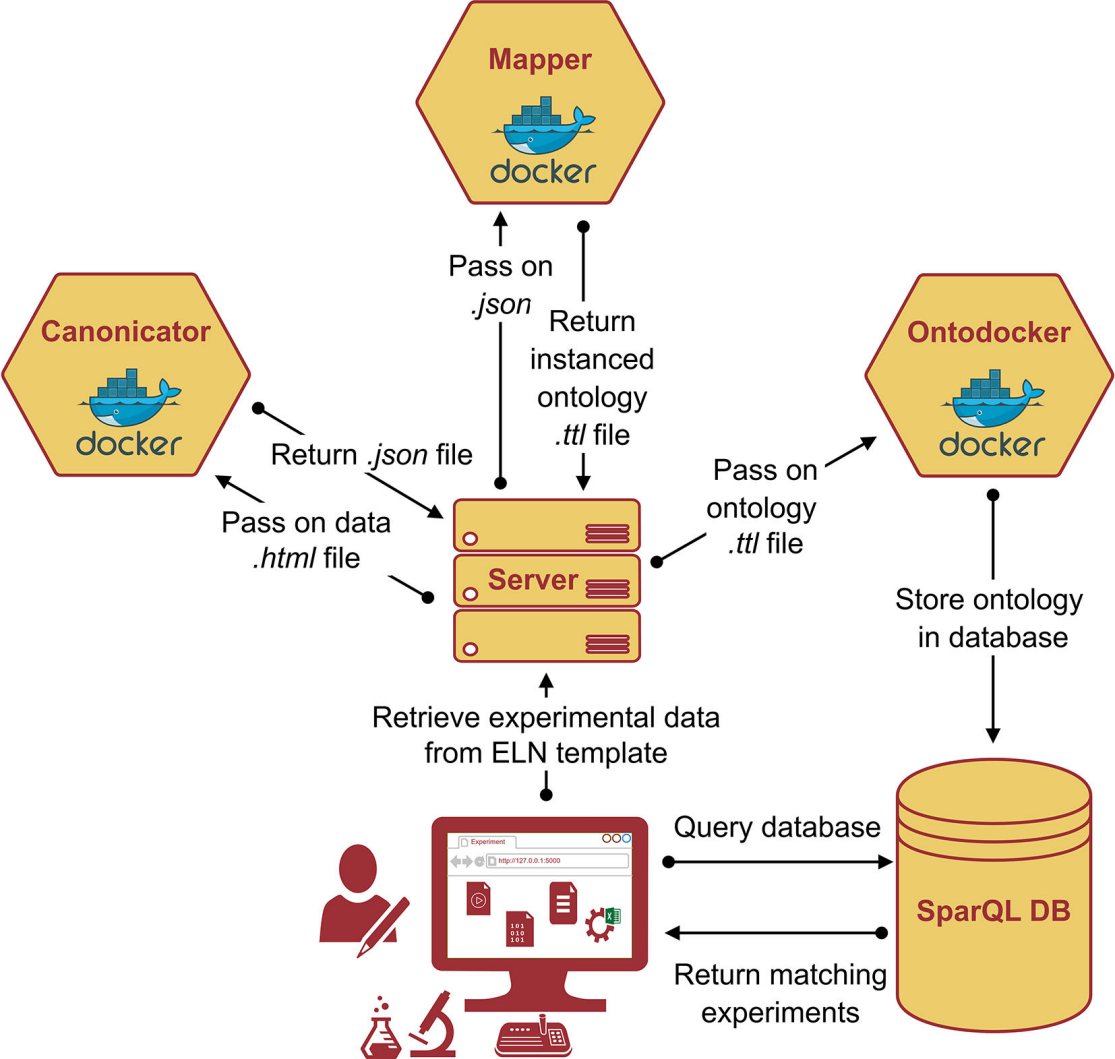
Schematic representation of the data acquisition pipeline (dap): Primary experimental data is retrieved from the ELN template in an *.html* format and converted the *.json* format using the *canonicator* microservice. The *.json* file is passed to the *mapper* microservice which returns the instanced ontology in *.ttl.* format (i.e., the A-box). The *ontodocker* stores the *.ttl* file in the SPARQL database which can be efficiently queried due to the semantic RDF triple representation of the experimental data.

The practical implementation of the AO requires a proper software service framework that processes the experimental data according to the complex ontological blueprint. To enrich the digital raw experimental data and transform it into FAIR research data, a *data acquisition pipeline* (dap) was designed. The *dap* is a collection of docker containers, or, more generally, microservices, that each supervises one step of data enrichment. The term ‘microservices’ originates from software development and refers to a design approach that breaks down tasks into multiple small, independent purpose-specific programs. These programs work together by communicating through application programming interfaces, rather than relying on a single application. This architecture enables seamless complementary integration into the established experimental workflows. The entire process is visualized in Figure 8: a central control unit, for example, a server distributes the tasks between the microservices. First, it retrieves raw experimental data from the Electronic Lab Notebook (ELN). This can be initiated, for example, by an experimenter clicking an “ennoble” button, or just be a (computer) job, that periodically checks for finished experiments. The raw experimental data were sent to the microservice c *anonicator.* The *canonicator* receives the experimental data, along with a set of instructions on how to transform the data, and returns a specific JSON file, called the canon-json, which contains the data using ontology terms. This aspect can be complex because it involves parsing human-written laboratory notes that do not necessarily follow a consistent structure or pattern. The canon-json file is already a serialization of the experimental data (an A-Box) and intrinsically contains knowledge of the application ontology (i.e. T-Box). However, it still needs to be parsed into an RDF file format, such as Turtle files (.ttl). This is the task of the microservice *mapper.* The mapper receives the canon-json and the ontologies (T-Boxes) for the experiment and produces the final A-Box, where each entry from the experimental raw data is represented as an *individual* of a *class* from the T-Box. Finally, the A-Box is sent to the microservice *ontodocker* which is a wrapper for a SPARQL (ontology) database. The *ontodocker* stores the A-Box dataset and handles querying (searching) the database.

**Technical maintenance**

Ontologies undergo constant advancements, and a change in a top- or mid-level ontology can lead to necessary adaptions in the application ontology. The necessity for technical maintenance is the reason for the many design choices made in the data acquisition pipeline ( *dap).* Such a framework is expected to run stably and, at the same time, adapt quickly to changes in experimental workflows and/or ontologies. This was achieved by decoupling the *dap* into three independent microservices. These microservices are static and remain unchanged, even if the input data or ontology is modified. However, the instructions for transforming data into the canon-JSON format need to be updated as necessary, which is why this software component is separated from microservices. This approach minimizes the modifications required for the *dap.*

**From lab notes to structured FAIR data**

**Figure 9.**
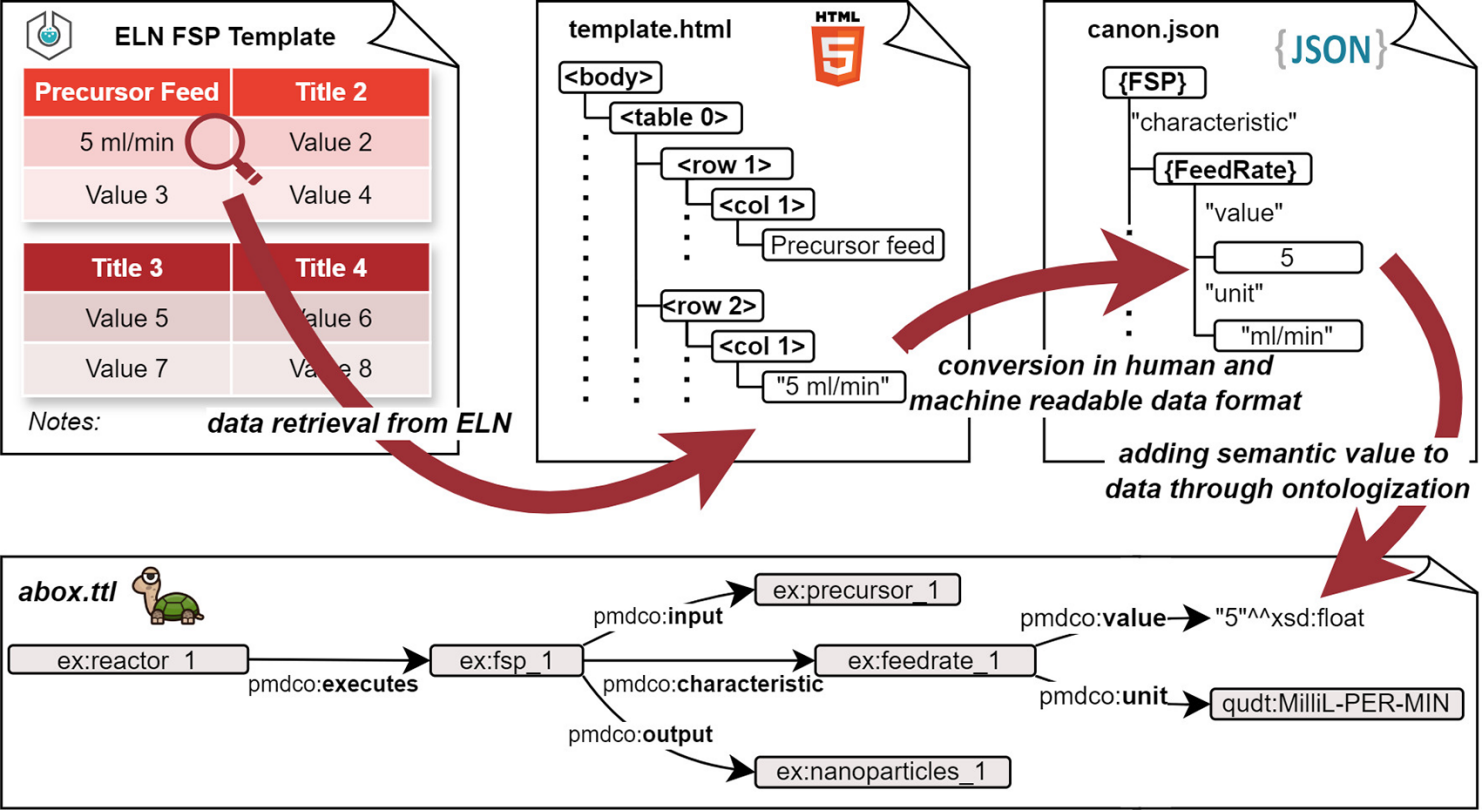
Exemplary initial data retrieval from the ELN FSP template and its parsing towards the semantic representation in the *abox.ttl* file.

The main difficulty in data enrichment is transforming the raw experimental data into structured data (the step involving the microservice *canonicator* is shown in Figure 8). The file formats and amount of raw data differed significantly between the different experiments. Generally, they can be divided into two categories: files produced by lab equipment (e.g., time series exports, automated reports) and (digital) lab notes written by humans. The former is easy to parse, but the latter inevitably forces researchers to adopt a (self-)prescribed consistent structure when documenting their experiments. For example, experimental documentation can be performed in the open source electronic lab notebook software eLabFTW. ^32^ The generated lab reports were essentially HTML documents. HTML, in itself, is a structured language, but the layout of the content can be freely chosen by the experimenter, for example, whether to use tables, paragraphs, or enumerations. To ensure consistent and stable parsing of the notes, this freedom must be restricted. One possible solution is to use a *template* for the experimental documentation. Templates are the built-in functionality of the eLabFTW. The template was selected to create a new entry for the experiment. It consists of empty tables in which the important process parameters are entered (cf. top-left Figure 9). For this specific template, and all the other files associated with the experiment, in this case, the FSP, a small custom Python module, is crafted. The module is imported by the *canonicator* and its functions are used to transform the raw data into the canon-json format (cf. the second arrow from the left in Figure 9). This “outsourcing” of program code has the huge benefit that only the template-module combination needs to be customized per experiment, while the *canonicator*, mapper and *ontodocker* can remain static, even if changes in the experimental workflow or the ontology occur.

There are certainly other ways to address the issue of parsing human-written data, such as parsing data using a Large Language Model (LLM). However, for the task at hand, the result of parsing needs to be exact and not probabilistic, and there simply does not exist enough data to sufficiently train such a model with the desired accuracy.

From a system design standpoint, the canon-json file may seem obsolete. However, in general, writing a parser that outputs JSON files is much easier than creating, for example, directly a Turtle file. Moreover, JSON is readable and comprehensible to humans, making potential mistakes and errors easier to identify during development and implementation. Thus, the canon-json file is situated between human-readable data and machine-readable data. Ultimately, a unified representation, such as the canon-json employed here, lowers the costs and effort of mapping to a different mid-level ontology if such a change becomes a requirement (e.g., migrating from PMDco2.0 to an emerging PMDco3.x). Provided the correct match of canon-JSON and T-Box, the mapper turns the data seamlessly into a Turtle file (cf. bottom Figure 9) and, thereby, performs the task of serializing the experimental data into an A-Box.

**Figure 10.**
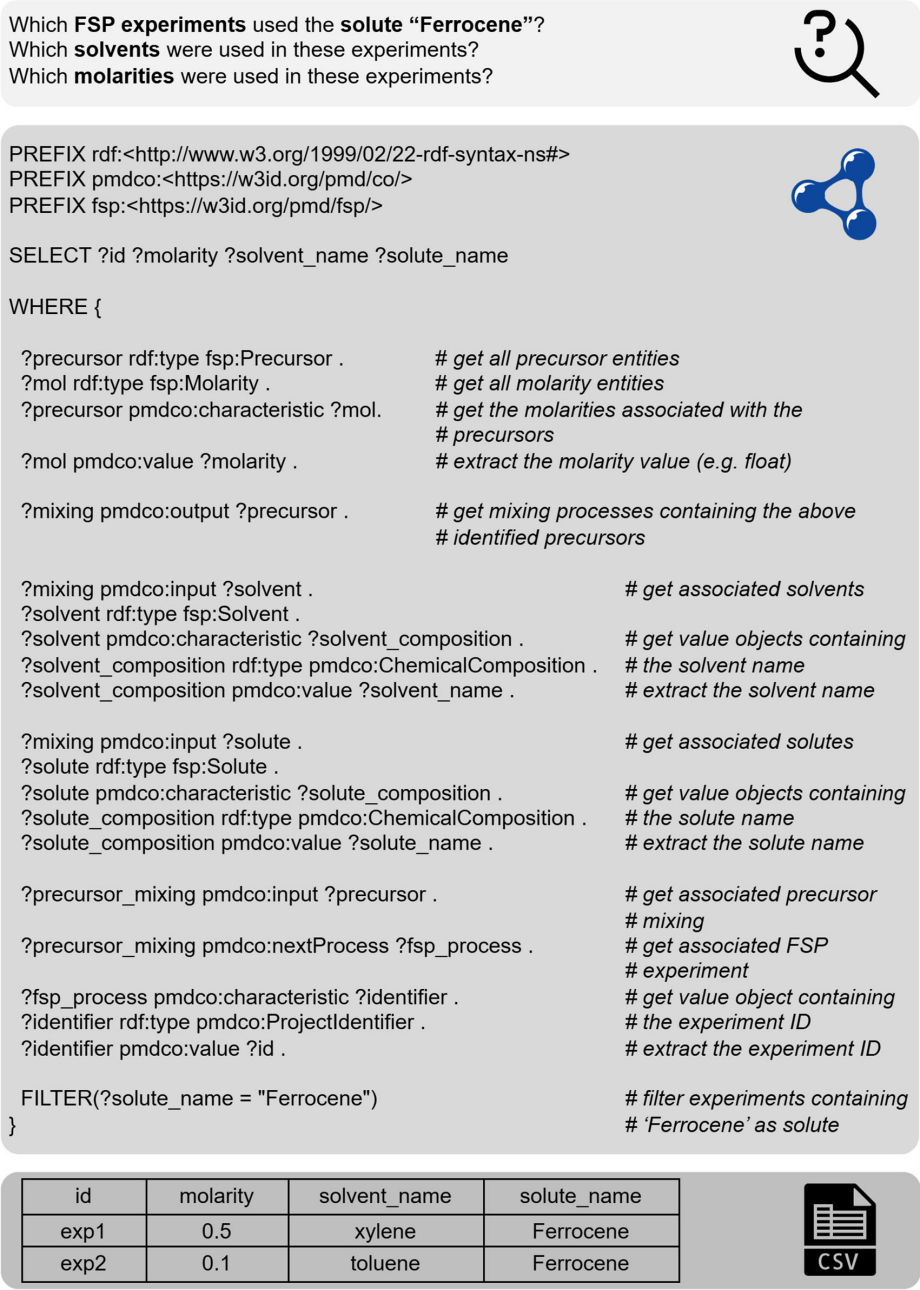
Demonstration of SPARQL querying: A dummy database with five experimental datasets, corresponding to five *.ttl* files, was queried to retrieve information of conducted experiments ( *?id*) with suitable solvents ( *?solvent_name*) and molarities ( *?molarity*) for the use of the solute ‘Ferrocene’ ( *?solute_name*) in the FSP process. The query output is summarized in a *.csv* file.

Once serialized and stored in the SPARQL database, the experimental data represented by the data triples can be efficiently queried, as shown in Figure 10. Here, the principal challenge of choosing suitable solute-solvent combinations for the precursor solutions in the FSP process ^33^ is addressed. In practice, this choice may require time- and resource-consuming solubility experiments, and from a user point of view, it is detrimental to transfer knowledge on both positive and negative solubility results to prevent redundancy. As a practical demonstration of the presented framework, a dummy database was queried to retrieve information on the solubility of the chemical ‘Ferrocene.’ The query was carried out using the local ontodocker [ 20] tool as an interface for the SPARQL database. In addition to querying, the Ontodocker tool provides an intuitive GUI and API to insert and update *.ttl* files (i.e., A-Boxes of experiments). The output of the SPARQL query is a *.csv* table that lists the requested information on matching entries, that is, the experimental ID and corresponding solutes, solvents, and molarity values. This example highlights the necessity of converting diverse experimental data into semantic datasets to ensure long-term findability, accessibility, interoperability, and reuse.

## Conclusion

The ongoing digitization in MSE provides new possibilities for retrieving undiscovered values from research data thanks to detrimental advancements in ML and AI, as well as steadily improving hardware and software. However, the incorporation of data science in research and industry requires profound (research) data management and long-term stewardship, with the aim of establishing standardized and interoperable high-quality data structures. Using semantic web technologies, namely ontologies, domain-specific concepts can be introduced to represent research data in a structured and harmonized fashion. These data structures become findable, interoperable, and reusable because of their semantically interconnected representations and machine actionability. Thus, the development of ontologies helps to adhere to FAIR data principles and improve data management, which is increasingly required by research funding institutions.

This paper presents the development of an application ontology for the flame spray pyrolysis process, which is adapted to the mid-level PMD core ontology. It serves as a use case that can be adapted for many MSE processes. The FSP AO and its concepts were based on published and established in-house experimental procedures and wording. Hence, the introduction of the proposed ontological FSP concepts is the first approach towards a common harmonized terminology and a comprehensive representation of experimental data, including precursor preparation and nanoparticle synthesis. The design of the FSP allows for efficient tracking of process characteristics and parameters to promote the reproducibility of results while reducing experimental redundancy. A data pipeline was developed to retrieve the original experimental FSP data from an electronic laboratory notebook and convert diverse datasets into a consistent semantic data format according to the ontological blueprint. Python-based microservices are used to ease maintenance and introduce the framework without disrupting the established lab workflows. Ultimately, a triple store database with a SPARQL interface aggregates the RDF triples, thus retaining knowledge from both published and unpublished data, while making it accessible for researcher queries.

The presented data pipeline shows a vivid example of the incorporation of semantic web technology in a laboratory environment. Owing to a standardized semantic representation of the research data, its management is eased and facilitates accessibility, reusability, and transfer of intrinsic knowledge. Specifically, the uniform documentation of primary data ensures ease of understanding and traceability for both experienced and inexperienced FSP users. Querying provides time- and resource-efficient support for mastering common process challenges. Furthermore, it aids in identifying existing knowledge and uncovering potential research gaps. In the long term, the consecutively growing SPARQL database is expected to play a pivotal role in future approaches to incorporating data science in FSP-related research endeavors.

## CRediT authorship contributions

**M.V.**: Methodology, Data curation, Writing - Original Draft, Visualization; **K.K.**: Methodology, Software, Data curation, Writing - Original Draft, Visualization; **J.G.**: Methodology, Software; **H.B.**: Writing - Review & Editing; **N.R.**: Conceptualization, Methodology, Writing - Review & Editing, Supervision, Project administration; **L.M.**: Conceptualization, Writing - Review & Editing, Supervision, Funding acquisition.

## Data Availability

All data are included in the repositories mentioned below in the Software availability.

## Footnotes

^1^

https://www.dfg.de/en/basics-topics/basics-and-principles-of-funding/research-data

^2^

https://erc.europa.eu/homepage

^3^

https://www.nfdi.de/

^4^

https://www.nist.gov/

^5^

https://dice.nims.go.jp/

^6^

https://emmc.eu/

^7^

https://www.materialdigital.de/

^8^

https://doc.elabftw.net/index.html

^9^

https://github.com/Leibniz-IWT/DataManagementGuidelines

^10^

https://flask.palletsprojects.com/en/3.0.x/

^11^

https://flask-restful.readthedocs.io/en/latest/

^12^

https://datacite-metadata-schema.readthedocs.io/en/4.5/

^13^

https://github.com/materialdigital/core-ontology

^14^

https://qudt.org/

^15^

https://www.ebi.ac.uk/chebi/

^16^

https://bioportal.bioontology.org/ontologies/NPO

^17^

https://www.w3.org/TR/PR-rdf-syntax/

^18^

https://protege.stanford.edu/

^19^

https://github.com/Leibniz-IWT/PMD-FSP-Ontology

^20^

https://github.com/materialdigital/ontodocker/tree/dev2

## Software availablility

Zenodo: Project ‘PMD-FSP-Ontology’: https://doi.org/10.5281/zenodo.14716872. ^34^

The project contains the following underlying data:

- PMD-FSP-Ontology.zip: The FSP application ontology, including its documentation, ELN template, and web demonstration; updated versions can be accessed in this repository: https://github.com/Leibniz-IWT/PMD-FSP-Ontology.
- ontodocker.zip: Docker file to perform SPARQL querys.
- Licence: BSD-3-Clause
