# Supplementary material for: An integrated data pipeline for semantic data representation of the flame spray pyrolysis process
Source: F1000Res. 2025 May 9;14:173. Originally published 2025 Feb 7. [Version 2] doi: 10.12688/f1000research.161252.2 (PMC12120422; doi:10.12688/f1000research.161252.2)
Supplement: Peer review history

# Reviewer report: Reviewer response for version 2

**DOI:** 10.5256/f1000research.181324.r384691

**Published:** 2025-05-28

**Authors:** Nick Garabedian

**Affiliations:** 1 Karlsruhe Institute of Technology, Karlsruhe, Germany

The paper presents a comprehensive framework for collecting FAIR research data from experiments using electronic lab notebooks (ELN) and ontology-based transformation. The paper is technically sound, and my comments focus on minor improvements aimed at enhancing clarity and accessibility for a broader audience.

 To increase the paper’s value, I suggest adding a brief, practical guide for typical users on how to produce FAIR data using this framework. Alternatively, clearly state early in the text that the intended audience is those familiar with the technical intricacies of FAIR data.

 I strongly agree with the authors' adoption of the "middle-out" approach, which is a balanced method for ontology development.

 The introduction provides relevant background but lacks a clear problem definition. What specific shortcomings or gaps in current initiatives (considering significant funding and multi-year projects) does this framework address? It would also be helpful to briefly discuss existing frameworks or projects aimed at producing FAIR data for experimental research and any known limitations of those approaches.

 Additionally, several informative background elements are scattered throughout the manuscript. Consolidating these within the introduction would significantly improve readability and coherence.

 Clarification would help regarding whether users are expected to create their own ontologies or if the initiatives described are responsible for developing and providing these ontologies.

 Minor consistency issue: "LabVIEW" is spelled inconsistently. Please standardize this.

 The integration of QUDT within the system requires additional detail. Given the importance and complexity of fully integrating Quantity Kinds and Units in a scalable manner, a more detailed explanation of the authors’ approach would be beneficial, especially highlighting how scalability challenges are managed.

 Overall, addressing these minor points would enhance clarity and broaden the paper's impact for diverse audiences.

Are the conclusions about the tool and its performance adequately supported by the findings presented in the article?

Yes

Is the rationale for developing the new software tool clearly explained?

Yes

Is the description of the software tool technically sound?

Yes

Are sufficient details of the code, methods and analysis (if applicable) provided to allow replication of the software development and its use by others?

Yes

Is sufficient information provided to allow interpretation of the expected output datasets and any results generated using the tool?

Yes

Reviewer Expertise:

FAIR Data, Experimental Materials Science.

I confirm that I have read this submission and believe that I have an appropriate level of expertise to confirm that it is of an acceptable scientific standard.

# Reviewer report: Reviewer response for version 1

**DOI:** 10.5256/f1000research.177257.r365507

**Published:** 2025-03-06

**Authors:** June W Lau

**Affiliations:** 1 National Institute of Standards and Technology, Gaithersburg, USA

This manuscript fills a critical knowledge gap in the research data ecosystem (i.e., I want to do this in my own research lab, how do I go about it, what are the considerations?). This manuscript gets us most of the way there, and I am in enthusiastic support of seeing it getting indexed. However, there are several points below where I believe the manuscript can be made substantially stronger and more impactful, and that it will be to the community's benefit if the authors can address my comments (and perhaps update the manuscript) before it is approved for getting indexed.

 ===================

**Section: Development of the FSP application ontology (page 6)**

 - First paragraph talks about top-down, bottom-up, middle-out approaches to onto development, and I think this is very worthy of expanding further. When I read these, the boundaries seem subjective. For example, in the context of the FSP framing, you can guide the reader through specifically what each of these three might look like. While I am not expecting this to be quantitative, it will help someone else get through a similar exercise for their own use case.

**Section: Capturing fundamental concepts (page 6-7)**

 Where you talk about mixing of two chemical species: Presumably there will be a chemical species ontology upstream of this that someone may elect to plug in. Later on, I see this is inherited from ChEBI ontology. We will come back to this comment later.

 -  (page 7 bottom) In the previous section, you talked about needing to develop an FSP ontology (and then spell out the recommended steps). Now you say it is best that this example (FSP) should align with what's available in the domain. This strikes me as a circular argument.

**Section: Serializing and validating the Ontology (page 9)**

 - The sentence with "Here, one must strike a balance between keeping the ontology as simple as possible and including all important details of the experiment." - Again, while I agree completely with this sentence, it would be far more help if the authors can give a specific example where this balance / compromise had to be made.

**Section: Key aspects of the pmdao_FSP (page 10):** This section can probably be shortened significantly compromising the text. There are other places in this manuscript where lengthier discussion would be more beneficial if space was a limit.

**Section: Modelling of general FSP process (page 12)**

 - About "pmdco:composed of object properties" - Just to be clear, and I want to make sure I am getting this correct: Solution = Solvent + Solute. The specific Solution, Solvent, and Solute are all Chebi individuals within the Solution, Solvent, and Solute subclasses. Therefore, when you use "pmdco:composed_of", you are saying some specific instance of Chebi "solution" is composed_of some specific instance of Chebi "solvent" + some specific instance of Chebi "solute". Am I correct?

**Follow-up question (and somewhat related to the ChEBI comment from the previous section on page 6):** Suppose you have a new typ of solution to add, you would then add this to the Chebi ontology, correct? Would you then also do a pull request to the Chebi onto to add the new substance? Or do you just keep it in-house?

 I guess what I am asking is, what is the reasonable effort (how much diligence should we give) to support FAIR? I would like to see a discussion of this.

**Section: Modelling of process parameters (page 12)**

 - About "(pmdco:input) and specified as the temporal volume flow..." - "Volume flow" is a good example of the kind of conundrum I have always had, and perhaps you can elaborate further in the manuscript. In this example, I image volume flow is actually two distinct value objects. In the first case, I think of volume flow as an input to the FSP reactor, like the knob or buttons on a panel. I think of these as the nominal values. In the second case, one might want to verify the volume flow with a measurement, and check that the measured and nominal values agree. How are these two cases distinguished in pmdco? Are they the same value object in both cases, and you designate the difference as one input, and one output? Or is the another way to signify that one is a measured value (and therefore deserves further treatments like uncertainty)?

**Section: Data ontologization using microservices (page 14) -** This paragraph here deserves to be a stand-alone paper, as there are lots of details within that would significantly benefit the MSE community. If this is an existing framework that is already working, I would appreciate a reference so that the reader man explore in greater depth.

Are the conclusions about the tool and its performance adequately supported by the findings presented in the article?

Yes

Is the rationale for developing the new software tool clearly explained?

Yes

Is the description of the software tool technically sound?

Yes

Are sufficient details of the code, methods and analysis (if applicable) provided to allow replication of the software development and its use by others?

Partly

Is sufficient information provided to allow interpretation of the expected output datasets and any results generated using the tool?

Partly

Reviewer Expertise:

Research data infrastructure

I confirm that I have read this submission and believe that I have an appropriate level of expertise to confirm that it is of an acceptable scientific standard, however I have significant reservations, as outlined above.

# Reviewer report: Reviewer response for version 1

**DOI:** 10.5256/f1000research.177257.r365506

**Published:** 2025-02-26

**Authors:** Leon Gorissen

**Affiliations:** 1 RWTH Aachen University, Aachen, Germany

**Summary**

 This article introduces a novel application ontology (AO) for the Flame Spray Pyrolysis (FSP) process, along with a data acquisition pipeline (DAP) that utilizes the presented ontology. The ontology is developed to address the absence of an existing application ontology specific to FSP, with the goal of ensuring semantic interoperability and aligning data generated in FSP processes with FAIR data principles.

 To demonstrate its practical application, a DAP is implemented for an exemplary FSP process, showcasing data reusability.

 The necessity of developing and publishing this ontology arises from the lack of an existing alternative. To maximize interoperability, the ontology builds upon standards and existing higher-level ontologies wherever possible. Similarly, the need for a new DAP stems from the requirement to integrate this ontology effectively. Where feasible, existing software solutions are leveraged. However, new software components were developed to parse data formats and conform them to the AO. Despite these efforts some design choices remain unexplained.

*The presentation of a novel AO presents an important contribution.*

**Weaknesses**

 Missing Version Information of ELN Software:

 A key issue is the absence of version information for the electronic laboratory notebook (ELN) software used. While the paper details the use of eLabFTW, it does not specify the exact version. This is critical because the DAP, more specifically the canonicator service, relies on HTML representations generated by the ELN software based on a template. According to the eLabFTW documentation, import / export functionality is only supported between instances of the same version [1]. Therefore, the version / SHA of the container image should be explicitly stated either in the paper or the accompanying repositories.

 Interaction with ELN Software:

 The implementation of a Flask-based web server with a GUI and RESTful API to upload data raises questions, as eLabFTW already offers both a web-based GUI and an API for data import. No justification is provided for why a custom implementation was necessary.

 Similarly, for data retrieval, the DAP, more specifically the canonicator service, relies on raw HTML data despite eLabFTW offering JSON export options via GUI, API, and CLI. Given that the canonicator service ultimately outputs JSON formatted according to the ontology, it is unclear why scraping HTLM was preferred over exporting JSON and creating a library for transforming arbitrary JSON data to fit the ontology. A rationale for the current approach should be provided or it is recommended to modify the canonicator to accept JSON input rather than HTML, to improve compatibility with different version or other ELN software. This design choice affects interoperability and should be addressed again.

**Major Problem**

 The ontology namespace ( https://w3id.org/pmd/fsp/) does not publicly resolve. Additionally, no corresponding pull request for this namespace could be found in the w3id repository, suggesting an issue with its publication. As a result, the ontology is not currently interoperable within the semantic web ecosystem. To enhance interoperability and ensure compliance with linked data principles, it is recommended to configure a persistent redirection for the namespace, allowing it to resolve to the ontology file or relevant documentation.

 [1] https://doc.elabftw.net/import-export.html

Are the conclusions about the tool and its performance adequately supported by the findings presented in the article?

Yes

Is the rationale for developing the new software tool clearly explained?

Partly

Is the description of the software tool technically sound?

Yes

Are sufficient details of the code, methods and analysis (if applicable) provided to allow replication of the software development and its use by others?

Partly

Is sufficient information provided to allow interpretation of the expected output datasets and any results generated using the tool?

Yes

Reviewer Expertise:

data pipelines

I confirm that I have read this submission and believe that I have an appropriate level of expertise to confirm that it is of an acceptable scientific standard, however I have significant reservations, as outlined above.
